# The impact of cannabidiol (CBD) in hyperglycemic zebrafish (*Danio rerio*)

**DOI:** 10.1371/journal.pone.0348975

**Published:** 2026-05-12

**Authors:** Elizabeth McCarthy, Leehy Gertner, Justin Ciocirlan, Victoria P. Connaughton

**Affiliations:** 1 Department of Biology, American University, Washington, District of Columbia, United States of America; 2 Center for Neuroscience and Behavior, American University, Washington, District of Columbia, United States of America; 3 Department of Anatomy, Physiology, and Genetics, Uniformed Services University of the Health Sciences, Bethesda, Maryland, United States of America; Noorda College of Osteopathic Medicine, UNITED STATES OF AMERICA

## Abstract

Diabetes Mellitus (DM) is a serious medical condition that impacts the lives of millions of people around the world. Complications of both micro- and macro-vascular nature occur later in life due to prolonged hyperglycemia. Hyperglycemia increases inflammation throughout the CNS and, more locally, in retina leading to the downregulation of tight-junction proteins at the blood retinal barrier and visual decline in Type 2 DM (T2DM) patients. Cannabidiol (CBD) is a cannabinoid known to lower inflammation and reduce blood glucose levels. These characteristics suggest that CBD could be used to mitigate hyperglycemic complications. To assess this, we examined if concurrent use of CBD (5 mg/L) during hyperglycemic induction would lower the risk for visual deficits in a zebrafish T2DM model. Using behavioral (optomotor response), molecular (RT-qPCR and Western Blot), and physiological (electroretinogram) techniques, we measured the response of CBD treatment on vision after a 4-week hyperglycemic period. After glucose treatment, zebrafish showed elevated blood sugar, reduced optomotor responses and compromised retinal electroretinogram recordings. Co-exposure with CBD increased performance on optomotor responses but did not significantly lower blood glucose levels. Glucose + CBD delayed photoreceptor a-wave and OFF-bipolar d-wave response times but did not restore the reduced b-wave and d-wave amplitudes observed in ERGs recorded from Glucose treated fish. Retinal homogenates from hyperglycemic fish with and without CBD co-exposure had decreased claudin-5 but increased occludin protein levels. Together, these results suggest that the CBD exposure protocol used here may broadly impact hyperglycemic sequelae but not specifically protect against microvascular complications.

## Introduction

Diabetes Mellitus (DM) is a serious metabolic condition that, as of 2021, impacted ~38.1 million US adults [1]. DM is characterized by an inability to regulate blood sugar levels, particularly the increase in blood sugar (hyperglycemia) that occurs after eating. DM can overall be described as a disease of insulin insufficiency or impaired insulin action [2]. Type 2 DM (T2DM) is characterized by an inability to regulate blood sugar levels due to impaired insulin action and accounts for 90–95% of all diagnoses [2,3].

Hyperglycemia caused by insulin insensitivity is a major risk factor for the onset and progression of the complications associated with T2DM. Diabetic neuropathy, nephropathy, retinopathy, and cognitive decline are the four main microvascular (small blood vessel) complications. Diabetic retinopathy (DR) is the most common of these complications and is the leading cause of blindness and visual impairments among the working-age population [4]. Patients with DR have an elevated chance to be diagnosed with diabetic nephropathy, neuropathy, and cognitive impairments [5–8], suggesting a common pathology across tissue types.

Hyperglycemia-induced retinal inflammation causes neuronal and vascular damage, increases blood retinal barrier (BRB) permeability, and triggers inflammatory pathways promoting the progression of DR. The increase in cell adhesion molecules reported in diabetic patients and animal models [9] causes leukocyte adhesion within retinal vessels prior to microaneurysms and capillary damage [10]. Hyperglycemic zebrafish [11] and 6-week-old T2DM Zucker Fatty Diabetic Rats [12] display increased retinal levels of inflammatory cytokines, some of which (IL-6, IL-1β, TNFα) are correlated with the severity of DR [9,13]. Elevation of MAPK (JNK) and NFκB pathways is also positively correlated with increased apoptosis and DR progression [12,13].

Cannabidiol (CBD) is a phytocannabinoid derived from the cannabis plant with anti-inflammatory properties. CBD acts by binding to, and activating, PPARα, PPARγ, and TRPV1 receptors [14,15]. CBD also targets the NFκB pathway by reducing phosphorylation of p38 MAPK, which decreases levels of inflammatory cytokines such as TNFα, IL-1β, IL-6, IFN-γ [16–18]. While this paper primarily focuses on the anti-inflammatory properties of CBD, it is important note that CBD also has broader modulatory effects such as anti-convulsant or antiepileptic, antidepressant, antipsychotic and antioxidant properties [15,19,20]. CBD treatment also helps reduce anxiety in humans [21], rodents [22,23], and zebrafish [24,25].

Previously, our lab used a zebrafish (*Danio rerio*) T2DM model to study the impact of hyperglycemia on retinal function. We have reported that 4-weeks of hyperglycemia causes thinning of the retina [26], reduces electroretinogram and red cone responses [27,28], increases retinal GFAP, IκB, and NFκB levels [11,28], reduces levels of the tight junction protein claudin-5 in retina [11,29], and changes optomotor responses [11,29]. Behavioral differences are apparent for up to 12-weeks of hyperglycemia, and recovery (return to normglycemic conditions) does not completely reverse these deficits [29].

Here, we employ a multistep approach to investigate whether a 20-minute co-exposure to CBD administered during prolonged hyperglycemia minimizes hyperglycemia-driven visual decline in zebrafish. We hypothesized that CBD administered during hyperglycemic insult would mitigate the visual decline and improve visual performance by reducing inflammation and the loss of retinal tight junction proteins.

## Materials and methods

### Animals

Adult wild-type zebrafish (*Danio rerio*) aged 4−12 months were obtained from either a commercial supplier (Live Aquaria, www.liveaquaria.com, or Carolina Biological, Burlington, NC), or bred in-house, and kept in the Aquatics Facility at American University. Zebrafish aged 4−12 months are considered adults as zebrafish reach sexual maturity between 2−3 months and old age by two years of age. Fish were held in an Aquatic Habitat (AHAB) (Pentair, Apopka, FL) system at ~28°C and on a 14−10hr light-dark cycle. Zebrafish were fed daily using commercial flakes (TetraMin^TM^, Blacksburg,VA). All fish were randomly chosen for participation and randomly separated into condition groups, with both males and females in each group. Upon the completion of the experiment, animals were anesthetized in 0.02% tricaine (tricaine-S, Western Chemical, Ferndale, WA) for 2 minutes (min) or until a lack of motor coordination and reduced gill movement were noted, at which point the fish were decapitated and tissue was collected for later investigation. All experimental procedures were approved by the Institutional Animal Care and Use Committee (IACUC) at American University (protocols #19−02 and 22−08).

### Hyperglycemia induction and CBD exposure

To induce hyperglycemia, zebrafish were placed in 2 L tanks maintained at 28–29°C. Fish were fed daily before transfers, at which time pH and temperature were recorded. Experimental groups were divided based on the *treatment* and the *exposure* regimen they received.

**Fig 1:**
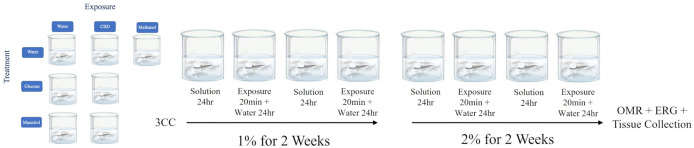
Overview of applied methods. Zebrafish were exposed in one of six *treatment + exposure* groups (left) over 4-weeks. For the first 2 weeks, a 1% Glucose (or Mannitol) *treatment* solution was used; for the next 2 weeks a 2% solution was used. CBD *exposure* occurred every other day when the animal was coming out of the *treament* solution (Glucose, Mannitol, or Water). After 4 weeks, behavioral recordings of optomotor responses (OMR) were obtained, followed by electroretinograms (ERGs) which analyzed retinal function. Retinal and liver tissue were then collected for qPCR and Western Blot analysis. CBD and Methanol (vehicle for CBD) were administered at a final concentration of 5 mg/ml and 0.005%, respectively.

*Treatment* refers to the type of solution the fish were maintained in over the 4-week period (i.e., Water, Mannitol, or Glucose). *Exposure* refers to what type of drug the zebrafish were placed into for 20 min every other day (i.e., Water, Methanol, or CBD). These parameters resulted in seven *Treatment + Exposure* groups: (1) Water + Water (stress control, n = 8), (2) Mannitol + Water (Osmotic Control, n = 6), (3) Water + Methanol (Vehicle Control, n = 19), (4) Water + CBD (Drug Control, n = 21), (5) Mannitol + CBD (Osmotic Drug Control, n = 21), (6) Glucose + Water (Hyperglycemic, n = 24), (7) Glucose + CBD (Experimental Group, n = 20) (Fig 1). Within the text we abbreviate these as Control (groups 1 and 2, not statistically different from each other for any parameter), Vehicle (group 3), CBD or Drug Control (group 4), Mannitol + CBD (group 5), Glucose or hyperglycemic (group 6), and Glucose + CBD (group 7). Glucose (D-glucose, #G8270) was purchased from Sigma (St. Louis, MO); mannitol (D-mannitol, #AC125340050) was purchased from ThermoFisher (Waltham, MA). CBD (C6395; #13956-29-1) was purchased from Millipore Sigma (Damstadt, Germany).

Hyperglycemia was induced using a stepwise alternate immersion protocol developed in our lab [30,31]. In brief, every day, during the same 3-hour time block, zebrafish were transferred from a tank containing one of the three *treatment* solutions (Water, Glucose, or Mannitol) to a tank containing only water (Fig 1). The subsequent day, the fish were transferred from the tank containing water to a new tank containing *treatment* solution (Water, Glucose, or Mannitol). For the first 2 weeks, the fish were maintained in a 1% solution (20 grams of Mannitol or Glucose dissolved in 2 L water). For the next 2 weeks, fish were maintained in a 2% (40 grams in 2 L water) solution, for a total *treatment* duration of 4 weeks [31]. Alternating immersion mimics the increase and decrease in blood sugar observed in diabetics, while the stepwise induction (1% to 2%) allows for a constant and steady increase of blood sugar [31]. After removing fish from a given *treatment* tank, the tank was cleaned and set-up for the next day. Solutions within the *treatment* tanks were the same temperature and photoperiod as stock tanks.

On the days when zebrafish were transferred out of *treatment* tanks, and before they were moved to water tanks, 20 min of drug *exposure* (Water, Methanol, or CBD) was administered (Fig 1). For each *exposure*, zebrafish, as either pairs or individuals were placed into a glass petri dish (3” diameter, 0.5’ deep) containing 50 mL of one of the *exposure* solutions (5 mg/L CBD or 0.005% Methanol or Water). Fish were allowed to swim freely for 20 min. After 20 min, the fish were placed back into water *treatment* tanks for 24 hr. CBD *exposure* solutions were made fresh from a stock solution every four exposure days and fresh system water was used for each exposure. To make the *exposure* solutions, 250 μL of Methanol (Vehicle; catalog #34860; Sigma) or the CBD stock (1 mg/mL) were mixed with 50 mL system water, resulting in a 5 mg/L (0.005%) *exposure* concentration. A 5 mg/L CBD dose was chosen based on the literature [25,32]. Blood sugar levels were the highest when *exposures* were performed (i.e., when the animals were removed from *treatment* tanks). This mimics a possible route of treatment for CBD as therapeutic drug vs a prophylactic drug.

### Optomotor response

Behavior was recorded on *treatment* day 28. To record the optomotor response (OMR) no more than two fish were placed in a 12-inch cylindrical glass dish that was set on top of a flat-panel computer monitor (Dell, Round Rock, TX), as in LeFauve et al., 2021 [33]. The monitor was connected to a Macbook Pro laptop (Apple, Cupertino, CA) located outside the behavioral chamber. The monitor and glass dish were housed in an all-black behavioral chamber, with a door, to prevent disruptions during recordings. Zebrafish were allowed to acclimate to the dish for 3 min and 30 sec before stimulus presentation. The stimulus, projected onto the monitor from the laptop, was a rotating black and white radial grating stimulus projected beneath fish [33]. The stimulus rotated first clockwise for 30 sec, followed by 30 sec of counterclockwise rotation, repeated twice. A gray screen was projected for 30 sec between each stimulus to act as both a control and a rest for the fish. Responses to the stimulus were recorded from above using a Canon (Melville, NY) video camera (VIXIA HFR700, 32x optical zoom, 57x advanced zoom, HD). Videos were scored, blinded to treatment, by watching the recording and counting the number of complete revolutions made by each fish during each stimulus presentation. A positive OMR occurred when the fish swam in the direction of the stimulus for one full rotation. Average values for each treatment group were compared.

### Electroretinograms

Four to eight retinal eyecups (2–4 fish) per group were prepared for ERG recordings. The remaining fish were sacrificed immediately, and brain, retina, and liver tissue were collected for later [11,28] use.

Following tricaine anesthesia, as noted above, fish were decapitated and blood sugar measurements were taken from the heart using a FreeStyle Lite Blood Glucose Meter (Abbot Diabetes Care, Alameda, CA). The eyes were then removed from the head and placed corneal side up on a piece of 0.45 µm black filter paper (#HABP02500; Millipore, Burlington, MA). Using Vannas Spring Scissors (#15044−08, Fine Scientific Tools, Foster City, CA), the lens and cornea were removed. The eyecup + filter paper were then transferred to the recording chamber and superfused with oxygenated MEM solution without glutamine (catalog #11090099; Thermofisher) equilibrated with 95%O_2_/5%CO_2_. A perfusion needle (28 gauge; catalog #MF28G67; World Precision Instruments, Sarasota, FL) was placed near the eye with a perfusion inflow rate of 0.3 ml/min (4 ml/min outflow), and a tungsten recording electrode (catalog #30031; FHC, Bowdoin, ME) was inserted directly into the vitreal space of the eyecup. The recording chamber was mounted on a fixed stage Olympus (Center Valley, PA) BX51WI compound microscope and imaged using an IR camera (Teledyne QImaging, Surrey, British Columbia) and Metamorph Imaging software (Molecular Devices, San Jose, CA).

Eyes were dark adapted in the chamber for 10–20 min. Retinal responses were evoked as in [34]. Specifically, a 300 msec white light pulse was administered at 7 irradiance levels ranging from ND 6.0 (dimmest) to ND 3.0 (brightest) in 0.5 increments. The white-light source was a 150 W Xenon arc lamp, imaged through UV compliant optics. Responses were amplified using a DAM80 amplifier (World Precision Instruments, Sarasota, FL), a bandpass from 0.1 Hz to 1 kHz, and a Digidata 1440A (Axon Instruments, Union City, CA) with a sampling rate of 2 kHz. Mean response component amplitudes and peak times were obtained from different treatment groups for the a-wave (photoreceptor response), b-wave (ON-bipolar response), and d-wave (OFF-bipolar response). ERG a-waves are a downward (corneal-negative) response immediately after light-ON; b-waves immediately follow a-waves and are a large corneal-positive response. Immediately after the end of the light pulse, a corneal positive d-wave was observed [34]. We determined differences in ERG responses by measuring differences in peak amplitude and implicit times for all three components, as in Jensen et al., 2025 [35]. Data were collected with pCLAMP 10 software (Molecular Devices, San Jose, CA) and analyzed using Origin 2021 (OriginLab, Northampton, MA). Graphs were made in Excel (Microsoft, Redmond, WA).

Each white-light protocol generated a total response dataset with 70, 4-trace-averaged ERGs, or 280 recordings. Each ERG (i.e., every eye) had 10 technical replicates at each of the 7-step irradiance level (ND 6.0 – ND 3.0). Our overall biological replicates (eyes), n = 3–8, per treatment group, generated 30–80 ERG traces at each brightness level. Technical replicates were removed from the analysis if the b-wave amplitude was negative or if the response occurred outside the appropriate interval. If more than five technical replicates had to be excluded from an eye, the eye was considered “dead” and was removed from analysis.

To conduct statistical analysis for ERGs, the 280 response waveforms from each eye were combined. Amplitudes (µV) and peak times (ms) for a-, b-, and d-waves were measured within fixed time intervals. ERG a- and b-wave components were measured in the interval 51–200 msec after stimulus onset. a-wave amplitudes were measured from baseline to the lowest point of the a-wave trough; b-wave amplitudes were measured from the lowest point of the trough to the peak of the b-wave response. ERG d-wave amplitude and peak times were measured in the interval 25–250 msec after stimulus offset [35].

### Molecular assays

#### RT-qPCR.

**RNA extraction:** RNA was extracted using the RNeasy Plus Mini Kit (Qiagen, Hilden, Germany) according to the manufacturer’s protocol for animal tissue. In brief, 1 brain or 2 combined retinas (roughly 25 mg of tissue) were lysed and homogenized in a solution of Buffer RLT Plus (Qiagen Kit) and 1% β-mercaptoethanol (catalog #M6250; Sigma). The lysate was spun through a gDNA Eliminator column (Qiagen Kit) whereby 1 volume of 70% ethanol was added to the flow-through, and samples were added to RNeasy spin columns (Qiagen Kit). A series of washes were performed with differing buffers, ending in RNA being elucidated in 40μl of RNAse-free water. Samples were quantified and stored at −80°C. Purity and concentration levels were quantified by measuring the A_260_/A_280_ absorbance ratios using a NanoVue Plus^TM^ spectrophotometer (Biochrom, Cambridge, UK). A_260_/A_280_ ratios between 1.9 and 2.2 were considered indicators of pure RNA and were selected for use. Gene-specific primer pairs (S1 Table) used for this study were designed using NCBI Blast (https://blast.ncbi.nlm.nih.gov/Blast.cgi) to span exon-exon junctions to avoid possible amplification of genomic DNA.

**RT-qPCR:** Isolated RNA was used in quantitative reverse transcription PCR (RT-qPCR) to measure the relative mRNA expression of the genes used. Reactions were performed using qScript One-Step SYBR green RT-qPCR, Low ROX Kit (Quantabio, Beverly, MA) in an AriaMx Real-time PCR system (Agilent, Santa Clara, CA). Reactions were prepared in a 96-well plate consisting of 10 μl of total volume. RNA was diluted using nuclease free water to total 15–25 ng. To create 9 μL of total reaction volume, 5 μL 2X One-Step Master Mix, 0.2 μL 1X qScript One-step Reverse transcriptase, 0.2 μL forward primer, 0.2 μL reverse primer, and 3.4 μL nuclease-free water were combined. To bring total reaction volume to 10 μL, 1 μL RNA was added to each well. The thermal cycling conditions consisted of the following: cDNA synthesis − 1 cycle/10 min/50°C, initial denaturation and Taq Polymerase activation − 1 cycle/5 min/95°C, denaturation − 40 cycles/10sec/95°C, annealing/extension − 1 cycle/30 sec/62°C. At the end of the cycling, a melt curve analysis was performed at 95°C for 1 min, 55°C for 30 sec, and 95°C for 30 sec. Dissociation curves were analyzed for primer specificity and absence of primer dimer formation.

Ribosomal protein L13A (*rpl13a*) was chosen as the housekeeping (hkg) gene because *rpl13a* has been previously identified as the most stable hkg in zebrafish for chemical treatment studies using qPCR analysis [36,37]. All samples were run in technical triplicate, and each plate contained all treatment groups (n = 2–7) (S1 Table).

RT-qPCR results were quantified using averaged cycle threshold (Ct) values from each technical replicate, which were determined after calculating a coefficient of variation (CV) for each qPCR reaction. CV was assessed for any inconsistency across replications. Comparative Ct values (ΔΔCt) were then determined for relative quantification of gene expression based on expression of the hkg gene. ΔΔCt values were then transformed to 2^ΔΔCt^ to obtain the expression fold change for each treatment relative to the water control. Statistics comparing biological replicates were run using ΔCt.

#### Western blot.

For changes in protein levels, frozen single brains (n = 3 fish) or pooled retinal tissue (n = 6–12 retinas from 3–6 different fish) were analyzed using Western Blots. Protein samples were isolated and centrifuged using an NP-40 lysis buffer (catalog #J60766.AP; ThermoFisher). A BCA Protein Assay (catalog #23225, ThermoFisher) was performed to determine the amount of protein in the sample. 20 μg protein by volume was loaded into each well. 25 μL was loaded into each gel lane by loading the correct ratio of NP-40:Protein, and 5 μL of 4X loading dye (NuPage #NP007). PageRuler^TM^ Plus prestained protein ladder (catalog #26620, ThermoFisher) was chosen to identify the proteins. The gel was transferred to a membrane using an iBlot 2 PVDF mini transfer stack (IB24002; ThermoFisher), and iBlot2 Gel transfer device (Invitrogen, Carlsbad, CA). The membrane was blocked in a 5% milk TBST solution (catalog #97062–370, VWR, Radnor, PA) for 30 minutes. Primary antibodies were then applied for either 1 hr at room temperature on a shaker table (80 rpm) or overnight at 4°C. Primary antibodies, and their dilution factors, were: claudin-5 1:500 (Invitrogen, Waltham, MA; #35–2500; RRID:AB_2533200), Occludin 1:750 (Invitrogen #71–1500; RRID:AB_2533977), JNK 1:1000 (Cell Signaling, Canvers, MA; #9252; RRID:AB_2250373), pJNK 1:750 (Cell Signaling #4668; RRID:AB_823588), AKT 1:1000 (Cell Signaling #9272; RRID:AB_329827), pAKT, 1:500 (Cell Signaling #9271; RRID:AB_329825), β-Actin (HKG) 1:1000 (Cell Signaling #4967; RRID:AB_330288).

Using TBST, the membrane was washed three times. A conspecific secondary antibody, diluted in 5% milk TBST, was applied and incubated for 45 minutes at room temperature. Claudin-5 received anti-mouse secondary antibody (Cell Signaling, 7076; RRID: AB_330924), while all other secondary antibodies were anti-rabbit (Cell Signaling, 7074; RRID:AB_2099233). All secondary antibodies were used at 1:2000 dilution. Using TBST, the membrane was washed an additional three times, after which the protein was stained with SuperSignal™ West Dura Extended Duration Chemiluminescent Substrate (ThermoFisher) for visualization. Blots were visualized using the ChemiDoc-It® Imaging System (UVP; Dallas, TX) with VisionWorks software.

Once the image was collected, the membrane was stripped using Restore^TM^ PLUS Western Blot Stripping Buffer (catalog #21059, ThermoFisher) before the next antibody was applied. Subsequent antibody exposure followed the above protocol. No more than four antibodies were used on a given blot.

Western blots were quantified using densitometry analysis in Image J software (https://imagej.net/ij/), and values were normalized to the β-Actin band. Fold change compared to Control (i.e., Water/Mannitol + Water) was calculated prior to statistical analysis. Each protein was considered its own dependent factor compared across the different groups.

### Statistics

For all analyses, differences across *treatment + exposure* groups were assessed using Kruskal Wallis nonparametric ANOVA followed by pairwise comparisons. Data was analyzed using SPSS software (IBM, ver. 26, 27). All p-values were evaluated at an α-level of 0.05. Because of the multiple pairwise comparisons, p-values were adjusted using Bonferroni’s correction. For all figures * < 0.05, ** < 0.01, and *** < 0.001.

## Results

### A 20-min CBD treatment altered liver cytokine levels and lowered stress levels

**Fig 2:**
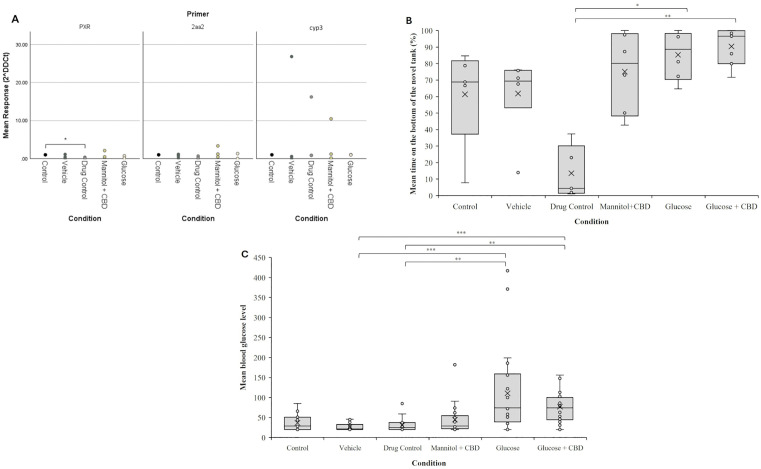
Validation of experiment parameters through liver cytochrome levels, novel tank assay, and blood glucose levels. **(A)** RT-qPCR results of liver tissue assessed for markers of liver metabolism using primers for *PXR, cyp2aa2 (2aa2)*, and *cyp3c1 (cyp3)* genes. Values were first normalized to housekeeping (hkg) gene *rpl13a*, and are graphed as fold change relative to Control, set as 1. Kruskal Wallis nonparametric ANOVA followed by pairwise comparisons revealed a significant decrease in *PXR* expression in livers from Drug Control fish compared to Control (*p* = 0.04; mean ± SE expression; 0.17 ± 0.09) There were no other significant differences between groups. Biological replicates n = 3 for every group. **(B)** Analysis of novel tank data showed Drug Control fish spent significantly less time (13.54 ± 7.19%) at the bottom of the tank compared to hyperglycemic zebrafish (*p =* 0.023; 85.3 ± 6.07% time at the bottom) and Glucose + CBD fish (*p* = 0.002; 90.36 ± 4.27% time at the bottom). **(C)** Four weeks of glucose exposure significantly elevated blood glucose levels. A Kruskal Wallis nonparametric ANOVA identified a significant overall effect of treatment on blood glucose levels (*p* < 0.001). Hyperglycemic and Glucose + CBD zebrafish had significantly higher blood glucose levels than Vehicle (*p =* 0.003) and Drug Control (p = 0.002) groups. Blood sugar levels of hyperglycemic fish were not different from blood sugar values of Glucose + CBD fish. Control n = 14, Vehicle n = 19, CBD/Drug Control n = 21, Mannitol + CBD n = 21, Hyperglycemia/Glucose n = 24, Glucose + CBD n = 20. Individual data points from biological replicates are shown in (A); whereas distribution across replications are presented as box and whisker plots in (B) and (C). * < 0.05, ** < 0.01, and *** < 0.001. Condition = *treatment + exposure* groups.

To confirm that a 20-min CBD exposure was sufficient for tissue uptake, we performed two analyses. First, we examined expression of cytochrome P450 (*cyp*) genes in liver homogenates collected from exposed fish. *Cyp* genes aid in liver detoxification and are involved in metabolism of CBD. *cyp3* is the most prominent gene in vertebrates [38], while *cyp2aa2* is dominant in zebrafish [39]. Pregnane X receptor (*PXR)* regulates the expression of these cytochromes [40]. CBD is an agonist of PXR receptors and, in humans, *cyp3a4* is a target gene of PXR [41]. RT-qPCR results identified significantly lower expression of *PXR* in CBD treated fish compared to controls (*p* = 0.04), with no change in expression for either *cyp3c1* or *cyp2aa2* genes (Fig 2A).

Second, we performed the Novel Tank Test as a behavioral analysis of CBD uptake [25]. Novel tank is a stress test in fish characterized by the fish staying at the bottom of the tank when first placed into the tank. How long the fish remains at the bottom and/or the time spent at the top of the tank are metrics used to identify stress responses. An increase in stress is characterized by more time at the bottom of the tank. Following 4-weeks of *treatment* and *exposure* conditions, CBD exposed fish spent an average of 14% of their time at the bottom of the tank, or 86% at the top of the tank (Fig 2B). This Novel Tank response of Drug Control (CBD exposed) fish was reduced compared to all other *treatment + exposure* groups, with significance observed compared to hyperglycemic zebrafish (*p =* 0.023), which averaged 85% of the time on the bottom, and Glucose + CBD fish (*p* = 0.002), which averaged 90% of the time on the bottom. Fish in Control and Vehicle groups spent between 75–80% of their time on the bottom of the tank. The results of this behavioral test identify an anxiolytic effect of CBD exposure, as previously reported in zebrafish [24,25] and indicate the exposure protocol was sufficient for compound uptake and efficacy.

### Elevated blood glucose levels were not significantly reduced by CBD treatment

There was an overall significant effect of *treatment* on blood glucose levels (*p* < 0.001). Hyperglycemic zebrafish had significantly higher blood glucose levels than Vehicle (p = 0.003) and Drug Control (p = 0.002) groups (Fig 2C). However, blood glucose values for the Glucose group were not significantly different from either the Glucose + CBD or Mannitol + CBD groups. Thus, after 4-weeks of *treatment*, blood sugar levels were significantly elevated in hyperglycemic fish (mean ± SE; 110.58 ± 21.39 mg/dL), consistent with our previous findings [26,31]. Fish co-exposed to Glucose + CBD had mean blood glucose levels of 77.15 ± 9.3 mg/dL, a trending difference.

### CBD co-exposure restored optomotor responses

**Fig 3:**
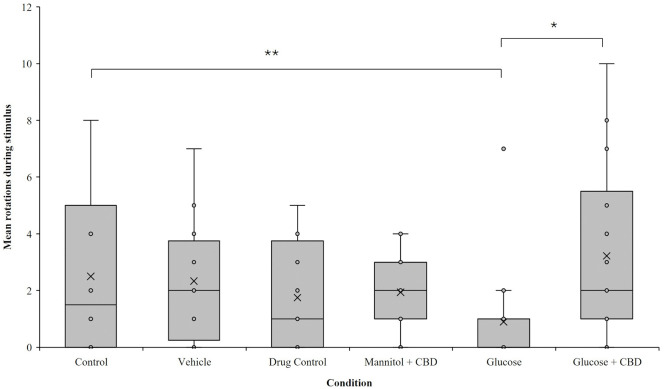
Reduced OMRs in hyperglycemic fish were prevented by co-exposure to CBD. Overall, a significant effect of *treatment* on the number of positive rotations completed during stimulus presentation was observed (*p* = 0.004). Hyperglycemic fish completed significantly fewer rotations than Glucose + CBD and Control (*p* = 0.023 and *p* = 0.006, respectively). Control n = 14 (3.73 ± 0.79 rotations), Vehicle n = 19 (2.17 ± 0.62 rotations), Drug Control n = 21 (1.86 ± 0.37 rotations), Mannitol + CBD n = 21 (2.18 ± 0.31 rotations), Hyperglycemic/Glucose n = 24 (0.78 ± 0.19 rotations), Glucose + CBD n = 20 (3.05 ± 0.65 rotations). Box and whisker plots are used to show the distribution of biological replicates. * < 0.05, ** < 0.01, and *** < 0.001. Condition = *treatment* + *exposure* groups.

Optomotor responses (OMRs) measured visual-based behaviors. There was a significant overall effect of *treatment* + *exposure* on OMR response (*p* = 0.004). Pairwise analysis revealed that the hyperglycemic fish completed fewer rotations in the direction of the stimulus compared to fish in the Glucose + CBD (*p* = 0.023) and Control (*p* = 0.006) groups (Fig 3). Thus, OMR performance was reduced in hyperglycemic zebrafish and co-exposure with CBD prevented this result.

### CBD co-exposure did not restore Glucose-induced changes in retinal ERG responses

**Fig 4:**
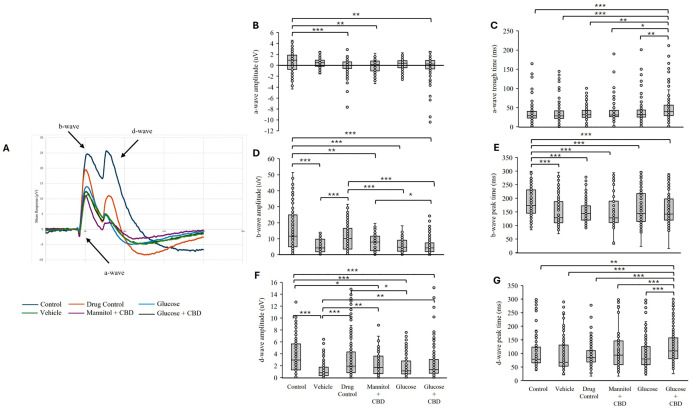
ERG peak amplitude and time to amplitude for a- b- and d-wave. **(A)** Mean ERG trace showing the photoreceptor a-wave, ON-bipolar cell b-wave, and OFF-bipolar cell d-wave components. **(B)** a-wave peak amplitude. Pairwise comparison showed significantly larger a-waves in the Glucose + CBD group compared to Control (*p* = 0.003). **(C)** Time to peak a-wave ERG response. Pairwise analysis showed a significantly delayed implicit time for the a-wave response between Glucose + CBD and all other treatment groups (Control *p* < 0.001, Vehicle *p* < 0.001, Drug Control *p* = 0.015, Mannitol + CBD *p* = 0.044, Hyperglycemia *p* = 0.020). **(D)** ERG b-wave peak amplitude response. Both Control and Drug Control groups had significantly greater b-wave amplitudes than all other treatment groups (Vehicle, Mannitol + CBD, Hyperglycemia, Glucose + CBD; *p* ≤ 0.001 for all). Glucose + CBD had a significantly lower b-wave amplitude than Mannitol + CBD (*p* = 0.046). **(E)** Time to peak b-wave ERG response. Responses from Control fish had significantly delayed b-wave response times compared to all other treatment groups (Drug Control, Vehicle, Mannitol + CBD, Hyperglycemia, Glucose + CBD; *p* ≤ 0.001 all). **(F)** ERG d-wave amplitude. The Control group had a significantly higher d-wave amplitude compared to all other treatment groups except Drug Control (Vehicle, Hyperglycemia, Glucose + CBD; *p* < 0.001 for all, Mannitol + CBD; *p* = 0.023). Hyperglycemic fish also had smaller peak amplitudes than the Drug Control (*p* = 0.013) **(G)** Time to peak d-wave ERG response. Glucose + CBD had significantly slower times to peak than all other treatment groups (*p* < 0. 001). Control n = 30, Vehicle n = 24, Drug Control n = 30, Mannitol + CBD n = 24, Hyperglycemia n = 35, Glucose + CBD n = 26. In (B) through (G) box and whisker plots show the distribution of biological replicates. * < 0.05, ** < 0.01, and *** < 0.001.

We previously observed a decrease in ERG b-wave amplitudes in hyperglycemic fish [28]. Here, we questioned whether the overall decreases in ERG responses could be mitigated by CBD co-exposure. Overall, we saw that Glucose *treatments* (both Glucose and Glucose + CBD) decreased ERG b- and d-wave amplitudes (Fig 4), indicating CBD co-*exposure* did not alter these ERG response components in Glucose treated fish.

#### Photoreceptor a-waves.

ERG a-waves measure the cone photoreceptor response at light ON. Examining a-wave amplitude and timing revealed significant differences in both parameters (amplitude: *p* < 0.001| timing *p* < 0.001) across all *treatment + exposure* groups. Individual pairwise comparisons revealed that the mean a-wave amplitude in the Glucose + CBD co-*exposure* group was significantly larger (more negative) than Control (*p* = 0.003); however, there was no difference in a-wave amplitude between the Glucose and Glucose + CBD groups (Fig 4A–4C). Timing of the a-wave trough was significantly delayed in the Glucose + CBD co-*exposure* group compared to all other treatments (p ≤ 0.04, Fig 4C). Thus, Glucose *treatment* alone did not change a-wave responses, but Glucose + CBD co-*exposure* delayed the a-wave response time.

#### ON-bipolar cell b-waves.

The most significant difference in ERG responses was observed for the b-wave. This represents the large depolarizing response of ON-bipolar cells in response to light [34]. We identified a significant effect of *treatment* + *exposure* on b-wave response amplitude (*p* < 0.001). Pairwise comparisons revealed significantly reduced b-wave amplitudes in both the Glucose (*p* ≤ 0.001) and Glucose + CBD (*p* ≤ 0.001) groups compared to the Control (Fig 4D). However, the b-wave amplitudes in these two groups were not significantly different from each other. ERG b-wave amplitude recorded from Glucose + CBD fish was also significantly different from CBD (Drug Control, *p* ≤ 0.001) and Mannitol + CBD (*p* = 0.046) *exposure* groups; b-wave amplitude in the Glucose group was reduced compared to amplitudes recorded from Drug Control fish (*p* ≤ 0.001). There was also a significant effect of *treatment* + *exposure* on the timing of the b-wave peak (*p* < 0.001; Fig 4E), with all treatment groups showing faster b-wave peak times compared to the Control (*p* ≤ 0.001).

Together, these results indicate hyperglycemic animals had decreased b-wave amplitudes consistent with our previous findings [28]. Glucose *treatment* also quickened the time to b-wave peak and *co-exposure* with CBD (Glucose + CBD) did not return these values to control levels.

#### Off-bipolar cell d-waves.

ERG d-waves represent the OFF-bipolar cell response. We observed a significant effect of *treatment* + *exposure* on d-wave amplitudes (*p* < 0.001) across all treatment groups (Fig 4F and 4G). However, there was no difference in d-wave amplitude between Glucose and Glucose + CBD groups (Fig 4F), though both were reduced compared to Control values (*p* ≤ 0.001). ERG d-wave amplitude in Glucose *treated* fish was also reduced compared to the Drug Control group (*p* = 0.013) and the d-wave amplitude in Glucose + CBD fish were reduced compared to Vehicle (*p* = 0.007) *exposed* fish. Similar to a-wave implicit times, Glucose + CBD delayed d-wave peak time compared all other groups (Fig 4G), including hyperglycemic fish (*p* < 0.001).

Thus, hyperglycemic fish receiving CBD had decreased d-wave amplitudes and delayed peak times compared to Drug Control fish that received only CBD, whereas hyperglycemia alone only decreased d-wave amplitude. This suggests impaired d-wave responses in Glucose + CBD zebrafish.

### Expression of inflammatory markers was not changed by treatment or exposure

**Fig 5:**
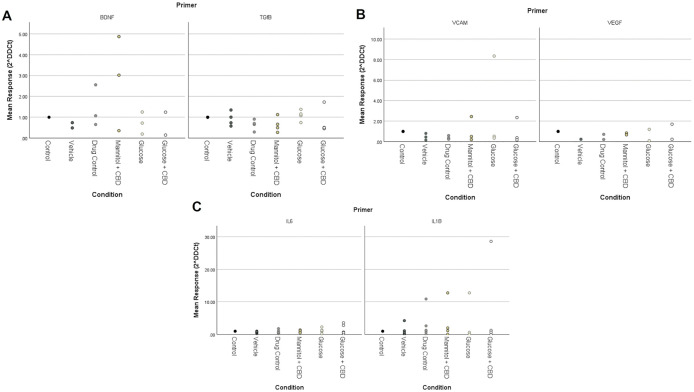
RT-qPCR results for retinal tissue markers of inflammation. Fold changes in expression for **(A)** retinal survival genes *BDNF* and *TGFβ*, **(B)** endothelial growth markers *VCAM* and *VEGF* and (C) inflammatory cytokines *IL6* and *IL1β* assessed in retinal homogenates. Expression was normalized to *hkg* gene *rpl13a* and fold changes are shown relative to Control, which was set as 1. Kruskal Wallis nonparametric ANOVA with pairwise comparisons was used to examine changes in expression for each gene. No overall significance across groups for any genes of interest were noted. Data presented show individual biological replicates. * < 0.05, ** < 0.01, and *** < 0.001. Condition = treatment + exposure groups.

We used RT-qPCR to examine differences in mRNA expression of inflammatory cytokines (*IL-1β, IL-6, TGFβ)* and endothelial cell growth markers *(BDNF, VEGF,* and *VCAM)* known to be affected by hyperglycemia and/or altered NfκB levels [13]. We found no statistically significant differences in expression of either inflammatory or endothelial cell growth markers across *treatment* + *exposure* groups in zebrafish retinal tissue (Fig 5).

### CBD co-exposure did not significantly affect protein levels

**Fig 6:**
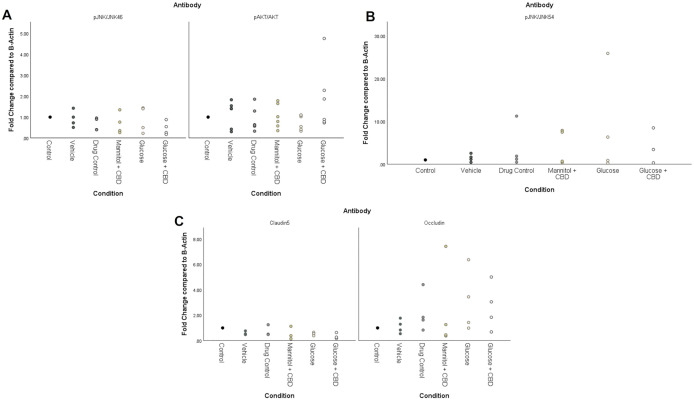
Western Blot analysis of inflammatory pathway and tight junction proteins. No statistically significant differences across *treatment + exposure* groups were observed for **(A)** phosphorylated JNK_46,_ phosphorylated AKT, or **(B)** phosphorylated JNK_54_ in retinal homogenates. However, tissue from the Glucose + CBD group did show an average 4x increase in pJNK/JNK_54_ (B) and a 2x increase in pAKT-AKT (A). Analysis of protein levels of the tight junction proteins claudin-5 and occludin **(C)** revealed opposite trends, with Glucose and Glucose + CBD fish showing an average upregulation of occludin and an average downregulation in claudin-5. Biological replicates for AKT, pAKT, pAKT/AKT n = 6. Biological replicates for Occludin, JNK_54_, JNK_46,_ pJNK_54_, pJNK_46,_ pJNK/JNK_54_, pJNK/JNK_46_ n = 4. Biological replicates for claudin-5 n = 3. Individual data points from biological replicates are shown. Condition = *treatment* + *exposure* groups.

Across treatment groups, there was no statistically significant effect of *treatment* + *exposure* for any target protein (pJNK/JNK, pAKT/AKT, occludin, claudin-5) in retina homogenates (Fig 6). Representative Western Blots are shown in S1 Fig. However, results with a fold change of ≥ 2x (strong upregulation) or ≤ 0.5x (strong downregulation) were considered meaningful increases or decreases in protein, respectively [42]. Glucose + CBD had an average 2x fold increase in pAKT/AKT protein (Fig 6A) and a strong upregulation (average 4x fold change) in pJNK/JNK_54_ expression (Fig 6B). Expression of a different JNK isoform, JNK_46_, was not changed by *treatment* or *exposure* condition (Fig 6A). The AKT pathway promotes cell survival, therefore an upregulation in this pathway could be protective against increased inflammation seen in the JNK_54_ pathway. Similarly, both hyperglycemic and Glucose + CBD fish show downregulated claudin-5 (<0.5x fold change), but upregulated occludin levels (average 2x fold change). An increase in protein levels of the tight-junction occludin could be acting as compensatory for the loss of claudin-5.

## Discussion

We assessed behavioral, molecular, and physiological endpoints of retinal function to determine the impact of CBD exposure on hyperglycemic complications in a zebrafish T2DM model. After 4-weeks of *treatment*, hyperglycemic zebrafish had significantly elevated blood glucose levels, as described previously [26,31]. CBD *exposure* was not effective in significantly lowering blood glucose levels compared to hyperglycemic fish. However, mean blood glucose levels were reduced in the Glucose + CBD group (Fig 2C), resulting in values similar to the Control, Vehicle, and Drug Control groups and identifying a trend in lowering blood glucose levels, as reported in the literature [43–45].

We have previously reported decreased OMRs in hyperglycemic zebrafish after up to 12-weeks of glucose treatment [29]. Our current results noted significantly decreased visual discrimination after 4-week of hyperglycemia in zebrafish, while Glucose + CBD showed significant protection against these visual deficits (Fig 3). To determine if the deficits observed in the OMRs were specific to the retina, we recorded ERGs. ERGs directly measure the responses of photoreceptors and bipolar cells in distal retina. As the OMR depends on the animal seeing the stimulus, we asked if the reduced positive OMRs in Glucose fish could be correlated with ERG changes (i.e., [46–48]) suggesting a retinal deficit. Previous work has reported reductions in a-wave, b-wave, and d-wave response amplitudes in zebrafish after 1-month of hyperglycemia [27,28]. Decreased b-wave amplitude was also reported in *pdx1*^*-/-*^ zebrafish, a transgenic model of Type 1 DM [49]. Consistent with these reports, we found decreased b- and d-wave amplitudes in hyperglycemic fish. However, decreased b- and d-waves were also observed in Glucose + CBD fish, suggesting CBD exposure did not help restore bipolar cell responses (Fig 4D and 4F). Further, implicit times for a-wave and d-wave components were significantly delayed in the Glucose + CBD, but not Glucose, fish (Fig 4C and 4G). Thus, ERG deficits in hyperglycemic fish were not rescued by co-exposure with CBD, as we observed for OMRs. Given this difference, it is not likely that a distal retinal (i.e., ERG) difference underlies the treatment-specific changes in behavioral OMRs. While OMRs require retinal processing (to see the stimulus), they also involve brain and spinal cord circuits [50]. Thus, the observed differences in physiological vs. behavioral outcomes could reflect the differential sensitivity retinal vs. non-retinal areas of the CNS (such as the brain and the spinal cord [51,52]) to CBD. The anxiolytic effects of CBD, for example, are attributed to interactions between CBD and serotonin (5-HT1A) receptors in brain [53]. We observed reduced anxiety in CBD treated fish (Fig 2B), with no change in total distance traveled (data not shown), suggesting a CBD-specific effect on individual brain circuits. Such a CBD-specific effect could be impacting circuits involved in generating the OMR. Hyperglycemia, therefore, may impact the broader CNS, over which CBD exposure may have a protective influence on generalized circuits.

We previously reported changes in NFκB (Rel-A) and GFAP protein in retinal homogenates after 4-weeks of hyperglycemia [11,28] suggesting inflammation. Here, we focused on other cytokines known to be altered by high blood sugar [54,55]. Surprisingly, we observed few statistical differences for any gene of interest across our *treatment + exposure* groups. In fact, examination of the fold changes in Glucose vs. Glucose + CBD groups (Fig 5), shows the same trends for four of the six genes examined (exceptions are due to the large outliers for *VCAM* and *IL1β* in Glucose and/or Glucose + CBD tissue), suggesting a similar outcome across groups. Similarly, comparisons of Control vs. Drug Control (i.e., fish receiving CBD or not) shows trending decreases in expression of *TGFβ, VCAM, VEGF*, and *IL6* in CBD-exposed fish, possibly reflecting a drug effect. We acknowledge that these differences only suggest interesting trends and that future studies with a larger sample size, and less variability, are needed. However, the results could also indicate that CBD has pathway- or cytokine-specific effects or that the inability of CBD co-exposure to significantly lower blood sugar levels prevents correction of gene expression changes triggered by hyperglycemia.

We assessed the efficacy of our CBD exposure protocol at both molecular and behavioral levels. Behaviorally, we observed that Drug Control fish spent significantly less time at the bottom of the tank during the Novel Tank Test (Fig 2B). This suggests that CBD was having an anxiolytic impact, as previously reported in zebrafish [24,25], rats [22], and mice [23] and indicating uptake of CBD by the fish in our *exposure* conditions. At the molecular level, we assessed expression of three genes in the liver, *pxr, cyp2aa2,* and *cyp3c1,* which are involved in metabolism/breakdown of potential toxins. In mammals, liver metabolism of CBD occurs by activating cytochrome 450 (CYP) enzymes such as CYP3A4 and CYP2C19 [56]. These enzymes, particularly *cyp3a4*, are downstream target genes of activated PXR [38] and CBD is an agonist of PXR [41]. Zebrafish liver also expresses *cyp3a* [40] or *cyp3c1* (according to GeneBank) and *cyp2aa2* [39], both of which are downstream of PXR activation [39,40]. Thus, we reasoned that CBD exposure should increase expression of *pxr, cyp3c1*, and *cyp2aa2* in zebrafish liver, indicating compound metabolism. However, we observed a decrease in *pxr* expression and no change in either *cyp2aa2* or *cyp3c1* in zebrafish liver homogenates (Fig 2A). We suggest three possible explanations for these results. First, the absence of an increase in *cyp* expression could be due to the short CBD exposure time. CBD uptake in zebrafish larvae peaked after 60 minutes of exposure with the subsequent decline attributed to CBD metabolism/excretion [24]. Our exposure duration of 20 minutes may not have been sufficiently long enough to induce changes in gene expression associated with CBD metabolism, though compound uptake occurred. Alternatively, CBD is reported to inhibit CYP3A activity in untreated mouse livers [57]. If CBD inhibition of CYP enzymes was occurring in our study, it could be represented as no change in gene expression. Finally, as an agonist of PXR, CBD binding PXR could have triggered a negative feedback pathway leading to reduced *pxr* expression and little or no changes in either *cyp3c1* or *cyp2aa2* expression levels. PXR is a nuclear receptor that forms heterodimers prior to binding to DNA response elements [58]. Reduced numbers of PXR, due to negative feedback, would reduce heterodimer formation which would inhibit subsequent gene expression.

DR progression upregulates the MAPK pathway(s), increasing phosphorylation of JNK and NFκB [12,13]. Based on our previous findings [11,28], we expected to observe changes in JNK protein levels. However, expression of pJNK/JNK_46_ was consistent across retinal homogenates from all *treatment + exposure* groups (Fig 6A). We also observed no statistical differences for either the pJNK/JNK_54_ isoform or pAKT/AKT (Fig 6A and 6B). However, pAKT/AKT levels were larger in the Glucose + CBD group compared to the Glucose group; while pJNK/JNK_54_ showed the opposite trend. This data suggests that AKT signaling (cell survival) is increased in Glucose + CBD conditions, whereas JNK_54_ signaling (inflammation) is increased in hyperglycemic retinas.

Inflammation induced by hyperglycemia compromises the BRB [59,60] by altering the availability of tight junction proteins [9,12]. We found overall higher levels of occludin protein (vs. claudin-5) in zebrafish retinal homogenates (Fig 6C), suggesting occludin may play a larger role in this tissue. Interestingly, occludin protein levels were observed to increase for both Glucose and Glucose + CBD fish, suggesting that CBD did not negate the impact of hyperglycemia on retinal occludin levels. Conversely, claudin-5 levels were reduced in both Glucose and Glucose + CBD fish (0.52x and 0.36x, respectively), suggesting CBD does not alter hyperglycemia-induced decreases in this protein. The opposite effects of hyperglycemia on these proteins could be due to their different roles within the junctional complex [61]. A decrease in claudin-5 may cause an increase in occludin to protect the integrity of the BRB [61]. The observed effects in our retinal tissue could be due to this compensatory mechanism triggered by hyperglycemic insult.

In summary, CBD significantly mitigated the hyperglycemia-induced visual deficits within the OMR task in Type 2 DM zebrafish, however, the impact of CBD exposure depended on the outcome measured. CBD co-exposure did not significantly impact protein levels of inflammatory markers nor did CBD exposure restore ERG deficits in b-wave and d-wave amplitudes observed in Glucose treated retinas. Likewise, both Glucose + CBD and Glucose treated fish showed similar changes to claudin-5 and occludin protein levels with the latter showing greater variation in responses. Therefore, we cannot conclude that CBD co-exposure was able to prevent the onset of visual deficits associated with hyperglycemia. Rather, we identify differential effects of CBD on hyperglycemia-associated behavioral vs. cellular outcomes. The overall increase in OMR score, lowered inflammation, lowered blood-glucose levels, and protected tight junction markers, suggest that CBD co-exposure could be considered to prevent the progression of microvascular complications.

## Limitations and future directions

While we tried to address the experimental constraints throughout the project, we can identify some limitations of the current work. First, cytokine expression was examined at the gene level at specific experimental time points. Cytokines may only be expressed for a short duration of time at the gene level, which may/may not correspond to our experimental timepoints, making differences and specificity hard to detect. Additionally, the mode of CBD administration may have been limiting. Bath administrations, over three trials, leads to unregulated uptake of CBD by the fish. Future studies with more chronic exposure or assessments immediately after exposure could resolve this issue. We also note uneven sample sizes across experiments. While our initial sample size was robust (based on a power analysis with an ANOVA design, 0.8 power, effect size of 0.5, and α = 0.05), these fish were subdivided for the molecular analyses, and we were not always successful in extracting enough pure RNA or protein from the retinas. Finally, another limitation is the use of the OMR vs. ERG analysis. While both assess vision, the ERG is a retina-specific analysis, while the OMR uses the retina as well as circuits in both the brain and the spinal cord. Some ERG responses were unexpected, such as the large variability in Control a-wave responses due to some positive values. It was also not uncommon to see fish swimming in the opposite direction of the OMR, though they also swam with the stimulus, suggesting more than one neuronal circuit is used for this response. A retina-specific behavior, such as the optokinetic response (OKR), would be a better behavioral correlate to the ERG. However, OKRs are difficult to do in adult animals because they require the animal to be immobilized during recordings.

Future experiments should continue to look at the different pathways implicated by inflammation such as the NFκB pathway and the p38 MAPK pathway. Furthermore, brain regions correlated to learning and memory, such as the lateral pallium and the telencephalon [62–64] could be examined for evidence of CBD recovery that might not be evident in other analyses. Given the wide range of effective CBD dosages studied in zebrafish [65], a higher dose could be considered. Overall, this research suggests that CBD may be a very powerful tool to prevent the progression of retinal microvascular complications associated with hyperglycemia, though the specific mechanisms are not yet uncovered.

## Supporting information

S1 Table Primer Pair sequences used for qPCRs. Each gene has a forward and backward primer associated with it. Each primer pair runs from the 5’ to the 3’ end. We had six genes of interest and one housekeeping gene (HKG). All genes were standardized to the HKG upon analysis.
(DOCX)

S1 Fig Western Blot analysis of inflammatory pathways. Representative Western Blots for (A) JNK, pJNK (B) AKT, pAKT and (C) occludin, claudin-5. Each blot contained all treatment groups, and each treatment group was run in biological duplicate. β-actin was used as the housekeeping protein on the blots and researchers normalized densitometry values to B-actin prior to analysis. Densitometry of blots was used to identify differences in protein levels.
(PDF)

S1 Video Representative optomotor response (OMR) video. OMRs were recorded by projecting a spinning pinwheel stimulus beneath the fish for 30 sec, followed by a white light stimulus for 30 sec. This sequence was repeated 3 times for a total recording duration of 3 minutes. While the fish could move with or against the stimulus, a positive OMR response was assessed by counting each complete rotation made in the same direction of the stimulus.
(MP4)
