# Supplementary figures and images for: The impact of cannabidiol (CBD) in hyperglycemic zebrafish (Danio rerio)
Source: PLoS One. 2026 May 12;21(5):e0348975. doi: 10.1371/journal.pone.0348975 (PMC13166913; doi:10.1371/journal.pone.0348975)

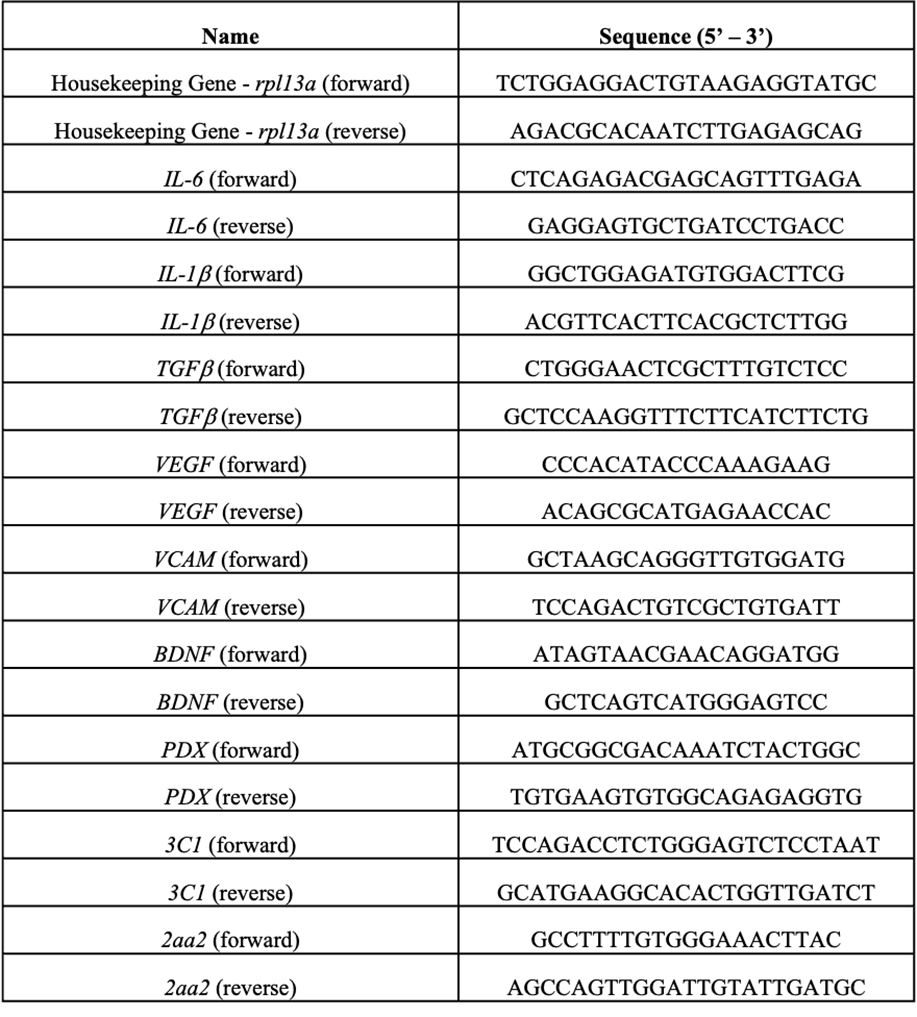

Supplement: S1 Table — Each gene has a forward and backward primer associated with it. Each primer pair runs from the 5’ to the 3’ end. We had six genes of interest and one housekeeping gene (HKG). All genes were standardized to the HKG upon analysis. (DOCX) [file pone.0348975.s001.docx]

**A**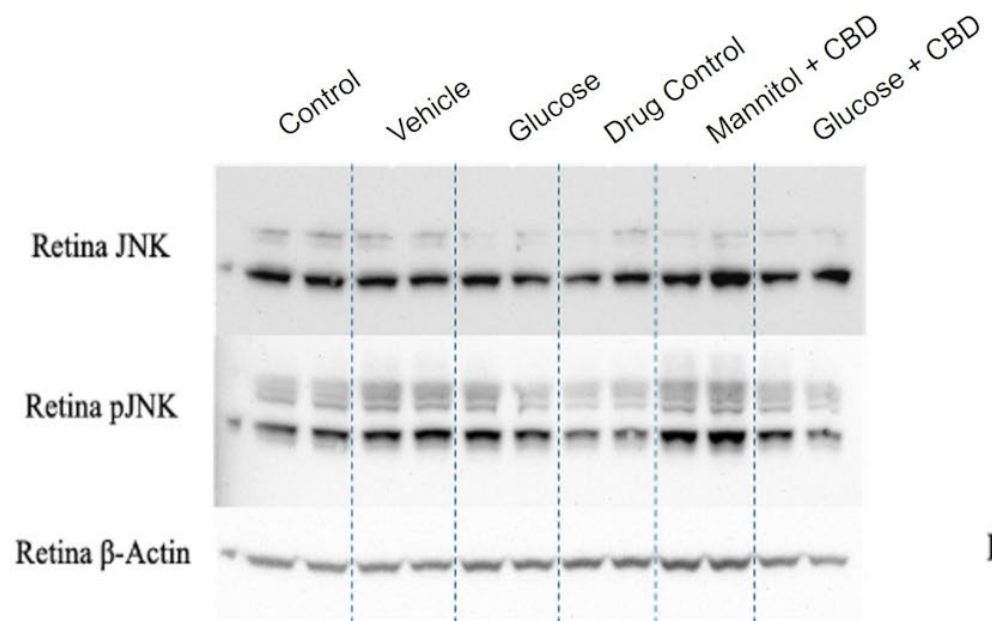**B**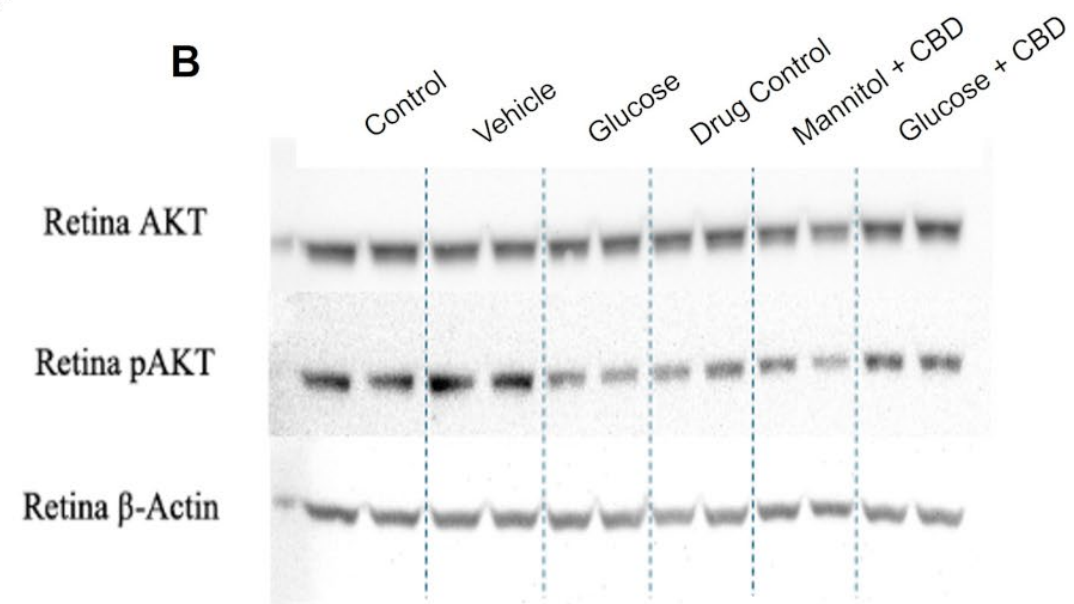**C**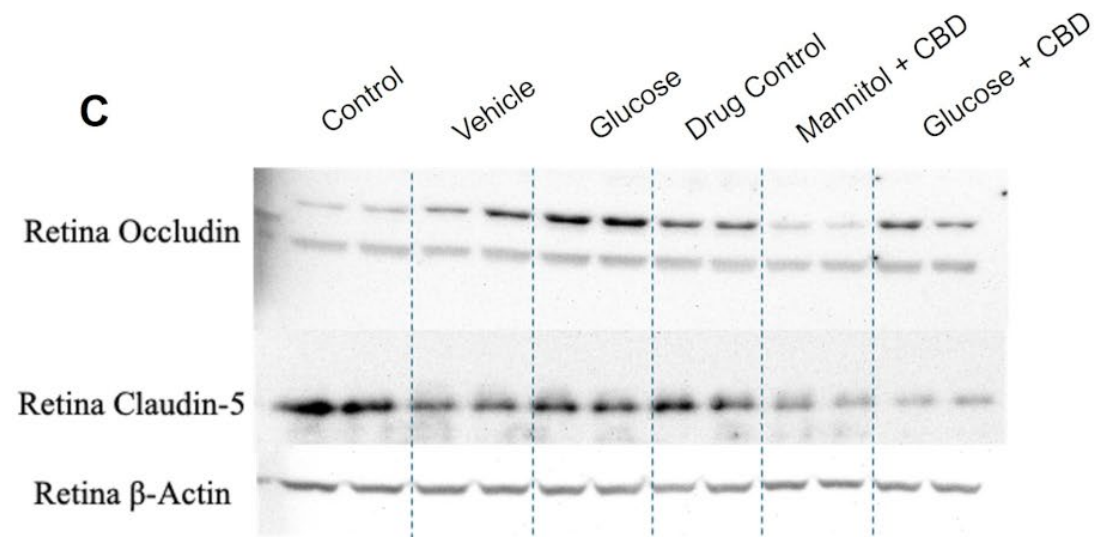

Supplement: S1 Fig — Representative Western Blots for (A) JNK, pJNK (B) AKT, pAKT and (C) occludin, claudin-5. Each blot contained all treatment groups, and each treatment group was run in biological duplicate. β-actin was used as the housekeeping protein on the blots and researchers normalized densitometry values to B-actin prior to analysis. Densitometry of blots was used to identify differences in protein levels. (PDF) [file pone.0348975.s002.pdf]
